# Polycistronic transcription of fused cassettes and identification of translation initiation signals in an unusual gene cassette array from *Pseudomonas aeruginosa*

**DOI:** 10.12688/f1000research.2-99.v3

**Published:** 2015-11-27

**Authors:** Érica L. Fonseca, Ana Carolina Paulo Vicente

**Affiliations:** 1 Laboratório de Genética Molecular de Microrganismos, Instituto Oswaldo Cruz, Rio de Janeiro, 4365, Brazil

**Keywords:** gene cassette, clase 1 integrons, fused gene cassette, transcription

## Abstract

The gene cassettes found in class 1 integrons are generally promoterless units composed by an open reading frame (ORF), a short 5’ untranslated region (UTR) and a 3’ recombination site ( *attC*). Fused gene cassettes are generated by partial or total loss of the *attC *from the first cassette in an array, creating, in some cases, a fusion with the ORF from the next cassette. These structures are rare and little is known about their mechanisms of mobilization and expression. The aim of this study was to evaluate the dynamic of mobilization and transcription of the *gcu14-bla*_GES-1_ */aacA4* gene cassette array, which harbours a fused gene cassette represented by *bla*_GES-1_ */aacA4*. The cassette array was analyzed by Northern blot and real-time reverse transcription-polymerase chain reaction (RT-PCR) in order to assess the transcription mechanism of *bla*_GES-1_ */aacA4* fused cassette. Also, inverse polymerase chain reactions (PCR) were performed to detect the free circular forms of *gcu14, bla*_GES-1_ *and aacA4*. The Northern blot and real time RT-PCR revealed a polycistronic transcription, in which the fused cassette *bla*_GES-1_ */aacA4* is transcribed as a unique gene, while *gcu14* (with a canonical *attC* recombination site) has a monocistronic transcription. The *gcu14* cassette, closer to the weak configuration of cassette promoter (PcW), had a higher transcription level than *bla*_GES-1_/ *aacA4*, indicating that the cassette position affects the transcript amounts. The presence of ORF-11 at *attI1*, immediately preceding *gcu14*, and of a Shine-Dalgarno sequence upstream *bla*_GES-1_/ *aacA4* composes a scenario for the occurrence of array translation. Inverse PCR generated amplicons corresponding to *gcu14, gcu14-aacA4 and gcu14-bla*_GES-1_/ *aacA4* free circular forms, but not to *bla*_GES-1_ and *aacA4* alone, indicating that the GES-1 truncated *attC* is not substrate of integrase activity and that these genes are mobilized together as a unique cassette. This study was original in showing the transcription of fused cassettes and in correlating cassette position with transcription.

## Introduction

Class 1 integrons are capable of inserting, excising and rearranging gene cassettes by a site-specific recombination mechanism. These assembly platforms can also act as expression systems due to the presence of a promoter region (Pc), which drives the expression of genes captured by integron ^1^. Moreover, naturally occurring integrons may have a second promoter (P2), which is activated by the insertion of three G residues between -35 and -10 hexamers ^1^. Gene cassettes are generally promoterless units associated with a recombination site ( *attC* or 59-be), which confers the ability of each structure to be mobilized independently ^2^, and the Left Hand (LH – 1L and 2L core sites) and Right Hand (RH – 1R and 2R core sites) domains from *attC* sites are crucial for this mobilization ^3^. Studies focusing on gene cassette translation in the context of integrons are rare, however, it was recently showed that *attC* sites regulate the translation of downstream cassettes due to their peculiar sequences composed by imperfect inverted repeats that form stem-loop structures. These secondary structures prevent ribosome progression throughout mRNA, reflecting in a decreased expression of more distal genes regarding Pc ^4^. Conversely, TIR (Translation Initiation Region)-deficient gene cassettes could have their expression promoted by the presence of ORF-11 ^5^. This ORF, when present, is found at the *attI* site preceding the gene cassette array. It codes for 11 amino acids and harbours its own Shine-Dalgarno (SD) sequence. Therefore, the ORF-11 recruits the ribosomes and, through an event of coupled translation, the subsequent TIR-deficient gene cassette could be expressed ^4, 5^. Gene cassettes can be found inserted in integrons or in other secondary sites, or free in the cytoplasm as a closed circle, in which the 5’ end (5’ UTR) and the *attC* recombination site are covalently linked ^6^.

As demonstrated previously, several stress conditions could evoke the activation of the SOS response resulting in integron-integrase expression ^7^. Therefore, under stress, the integrase activity increases, favoring the occurrence of integration/excision/rearrangements events.

Although rare, fused cassettes may be generated by partial or total loss of the first *attC*, retaining both complete coding regions and, therefore, creating permanent gene arrays comparable to bacterial operons ^8^. The functionality of such structures has been indirectly inferred by the resistance profile of transformants carrying the fusion ^9^; however, the transcription itself has never been verified.

This study showed the dynamics of fused cassette mobilization, the co-transcription of the *gcu14*- *bla*_GES-1_/ *aacA4* cassette array and the effect of cassette position on transcription levels in *Pseudomonas aeruginosa* wild lineages carrying class 1 integrons. Moreover, the presence of translation signals in this gene cassette array was determined.

## Material and methods

An unknown Open Reading Frame (ORF), *gcu14* ( gene cassette of unknown function), followed by the fused cassette *bla*_GES-1_/ *aacA4*, created by partial loss of GES-1 *attC* were present in integrons from clinical *P. aeruginosa* isolates (PS1 and PS26) ^10^. Total RNA was extracted and purified according to the manufacturer’s instructions with the SV 96 Total RNA Isolation System (Promega). Northern blot using 7 μg of total RNA from PS1 and PS26 was performed in order to detect the transcript originated from *gcu14*- *bla*_GES-1_/ *aacA4* cassette array. After electrophoresis in a denaturing-formaldehyde 1.5% agarose gel, the total RNA was transferred to the Hybond-N ^+^ nylon membrane (GE Healthcare) by upward capillary transfer. An amplicon of 519bp corresponding to part of the *bla*_GES-1_ gene was used as a probe ( Table 1) in hybridization assay. The GES probe was labeled with the AlkPhos Direct Labelling kit (GE Healthcare) and hybridized with the target RNA immobilized on the Hybond-N ^+^ membrane as recommended. The chemiluminescence was detected with the *CDP*-Star detection reagent (GE Healthcare) according to manufactures. Immediately after applying the detection reagents, the blot was drained, incubated five minutes at room temperature and exposed to the Hyperfilm ECL (GE Healthcare) for 60 minutes at room temperature.

**Table 1.** Primers used in conventional, inverse and real-time PCR reactions.

Primer	Primer sequence (5' – 3')	Size (bp)	Target
**Primers for conventional PCR**			
Ges F Ges R	GCGTTTTGCAATGTGCTC CCAGTTTTCTCTCCAACAACC	519	Internal fragment of *bla*_GES_ gene
**Primers for inverse PCR**			
Gcu14 FSQ Gcu14 RSQ	AGCAATCAACACACAGGGG CTCGCGTAAATGCACCGCTT	130	*gcu14* circular form
GES FSQ GES RSQ	CAAGTTATTACACAACTCAT AGTGCGTGAATGAAGCGCAT	110	*bla*_GES-1_ circular form
AACA4 FSQ AACA4 RSQ	GCCAGGCATTCGAGCGAACAC ATTTAGCCACTCACATAGAGC	188	*aacA4* circular form
**Primers and probes for real time PCR (TaqMan)**			
RpsL F RpsL R Probe	GCCTGCGCTGCAAAACT TTTCGGCGTGGTGGTGTAT TCGTGGCGTATGCACC	67	*rpsL* transcripts
Gcu14 F Gcu14 R Probe	CATGCGCTTCTTGGTTCGT ACGCCAGCTTGGATGCAA ATGCCACGAGACCTT	56	*gcu14* transcripts
Ges F2 Ges R2 Probe	GTGCAGCTTAGCGACAATGG CACAGAGTCGCCAATTTTACGA AATTGCAGCAGGTCCGCC	99	*bla*_GES-1_ transcripts
AacA4 F AacA4 R Probe	CAAGCGTTTTAGCGCAAGAGT TCGGCTCTCCATTCAGCATTG CCGTCACTCCATACATTG	59	*aacA4* transcripts
Ges F3 AacA4 R2 Probe	TCCTGAGCACGGACAAATAG TCATAGAGCATCGCAAGGTC TTCCGTCACACTGCGCCTCA	134	Polycistronic transcripts

In order to verify whether the relative position of gene cassettes on the variable region plays a role in transcription level, real-time RT-PCR reactions using the TaqMan System (Applied Biosystems) were performed with primers and probes detailed in Table 1. The *rpsL* gene of the *P. aeruginosa* chromosome was amplified by PCR ( Table 1) and used as a reference gene for normalization. The relative quantification (RQ) results were presented as ratios of gene transcription between the target gene (cassettes) and the reference gene ( *rpsL*), which were obtained according to the following equation: RQ=2 ^-ΔCT^, where CT is the value corresponding to the crossing point of the amplification curve with the threshold and ΔCT=CT target gene minus CT reference gene. The effect of cassette position on gene transcription was considered significant when the ratios obtained between RQ values (RQ value of cassette 1/RQ value of cassette 2) were ≥2.0, taking into account the standard deviation intervals.

In order to induce cassette excision from integrons, PS1 and PS26 strains ^10^ were submitted to thermal stress during the log growth phase to induce integrase activity. Cells were grown on Luria-Bertani (LB) broth medium (OXOID) at 37°C for two hours. Subsequently, the bacterial cultures were submitted to a heat shock at 4°C for 30 minutes and immediately incubated at 42°C for another 30 minutes. Briefly, the total DNA from PS1 and PS26 cultured under thermal stress were obtained with the Wizard Genomic DNA purification kit (Promega) following manufacturer recommendations and used as templates in inverse PCR reactions. The inverse PCR was performed with primers facing outwards towards the ends of *gcu14*, *bla*_GES-1_ and *aacA4* so that only circular gene cassette configurations would be amplified. The reactions targeting the circular forms of *gcu14*, *bla*_GES-1_, *aacA4* and *bla*_GES-1_/ *aacA4* fusion was performed with primers and combinations described in Table 1. The inverse PCR was performed using Platinum *Taq* DNA Polymerase reagents (Invitrogen), and the following components were added to a sterile 0.2-mL tube: 5 µL of 10X PCR buffer (1X final concentration); 1 µL of 10mM dNTP mixture (0.2 mM each); 1.5 µL of 50mM MgCl _2_ (1.5 mM final concentration); 2 µL of 15 µM of each primer (30 µM each); 100 ng of template DNA; 0.3 µL of Platinum *Taq* DNA Polymerase (1U final concentration). The tubes were incubated in the Eppendorf MasterCycler (Eppendorf) at 94°C for 2 minutes and PCR amplification was performed in 40 cycles consisting of: 94°C for 30 seconds; 55°C for 30 seconds; and 72°C for 3 minutes. The amplicons generated with the inverse PCR were purified using Wizard SV Gel and PCR Clean-Up system kit (Promega) and directly sequenced on both strands. Sequencing reactions were performed with Big Dye Terminator RR Mix (Applied Biosystems) in an ABI 3730 XL DNA Analyzer (Applied Biosystems). Nucleotide sequences were compared to those available in the GenBank database accessible on the National Center for Biotechnology Information website ( http://www.ncbi.nlm.nih.gov). All primers used in PCR, sequencing and real time RT-PCR are described in Table 1.

Analyses *in silico* were performed to search for a potential promoter for *gcu14* gene cassette. The 5’UTR from *gcu14* were submitted to the promoter predictor programs Neural Network for Promoter Prediction version 2.2 (Berkeley Drosophila Genome Project, http://www.fruitfly.org/index.html) and BPROM (SoftBerry, http://linux1.softberry.com/berry.phtml). Results with the highest scores were selected as candidates for a putative promoter.

## Results and discussion

**Figure 1.**
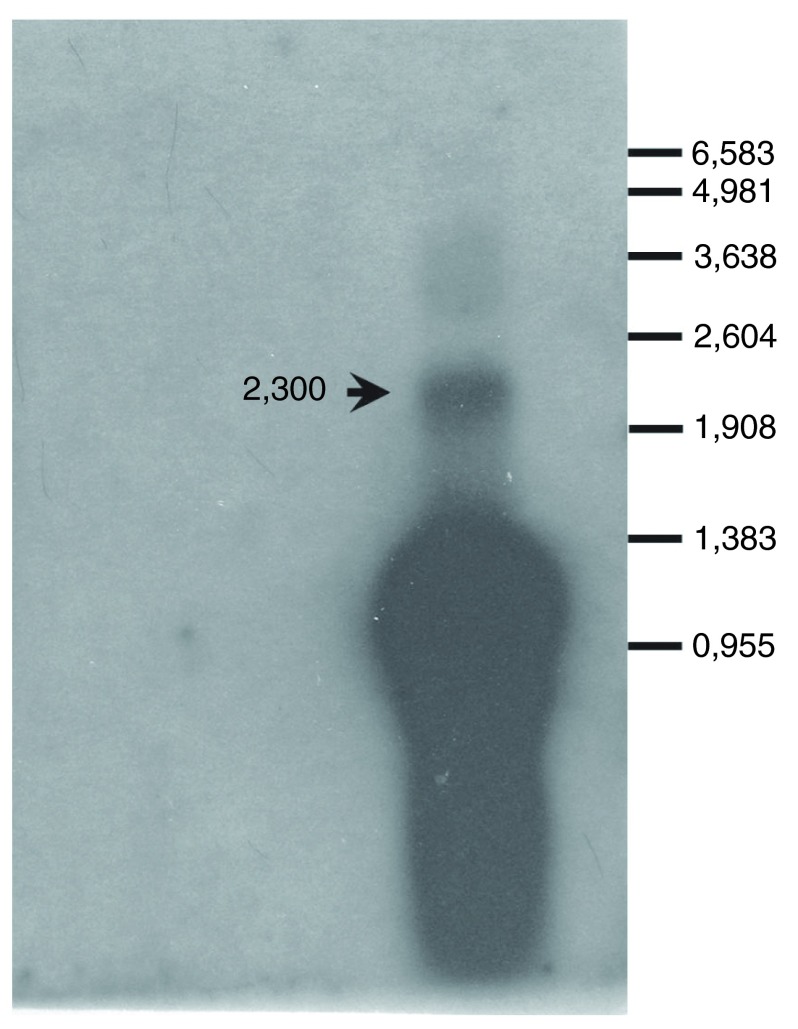
Northern blot analysis of *gcu14*- *bla*_GES-1_/ *aacA4* gene cassette array from *P. aerginosa* class 1 integron. The full length transcript (2,300 bases), corresponding to the entire gene cassette array, hybridized with the GES probe (arrow). The fragment sizes of the RNA marker (Promega) used in RNA electrophoresis are indicated.

The integrons analysed in this study harbored the weak Pc configuration (PcW) ^11^, and they carry the *gcu14* as the first gene cassette (see reference 12 for nomenclature), which has not been reported so far. Considering that transcription initiates from the Pc promoter placed upstream the cassette array, both monocistronic and full length polycistronic transcripts could be identified. In fact, Northern blot and hybridization assays revealed a unique signal of approximately 2,300 bases, which corresponds to the co-transcription of the entire array ( *gcu14*- *bla*_GES-1_/ *aacA4*) ( Figure 1). This result is in agreement with previous work in which the occurrence of transcripts containing more than one gene cassette was observed by Northern blot analysis ^1^. Moreover, this finding gives support to the lack of *attC* function in terminating transcription of downstream gene cassettes as demonstrated previously ^4^.

**Figure 2.**
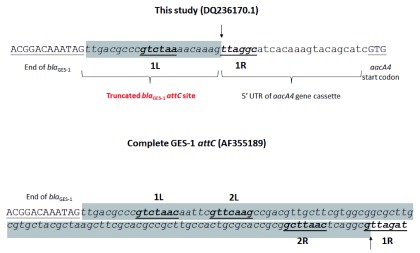
Sequences of the canonical (AF355189) and the truncated (this study) GES-1 *attC* sites. The underlined uppercase letters represent the *bla*_GES-1_ and *aacA4* partial coding regions. Italicized lowercase letters highlighted in grey define the *attC* sites, and their core sites (1L, 2L, 2R, 1R) are in boldface and underlined. The vertical arrows show the recombination point and the beginning of the next cassette.

This fusion retained both entire coding regions and, due to a possible erroneous recombination event, the *bla*_GES-1_ *attC* site was replaced by part of the *attI1* site ( DQ236170) ^10^, reducing it to the 6bp from the 1L core site ( Figure 2). Taking into account that the region responsible for stem-loop formation was missing in GES-1 *attC* and the participation of this site in terminating translation ^4^, our findings indirectly suggested that *bla*_GES-1_ and *aacA4* translation is occurring in a unique step.

The strains were submitted to thermal stress conditions in order to verify the dynamics of mobilization of *gcu14*- *bla*_GES-1_/ *aacA4* gene cassettes. Since the excision event depends on the recognition of the LH and RH domains of the *attC* site, and that the 2L core site and the entire RH domain are missing in GES-1 *attC* ( Figure 2), it is expected that the *bla*_GES-1_/ *aacA4* excision occurs only at the *aacA4 attC* site, and that this structure is excised together as a unique cassette.

**Figure 3.**
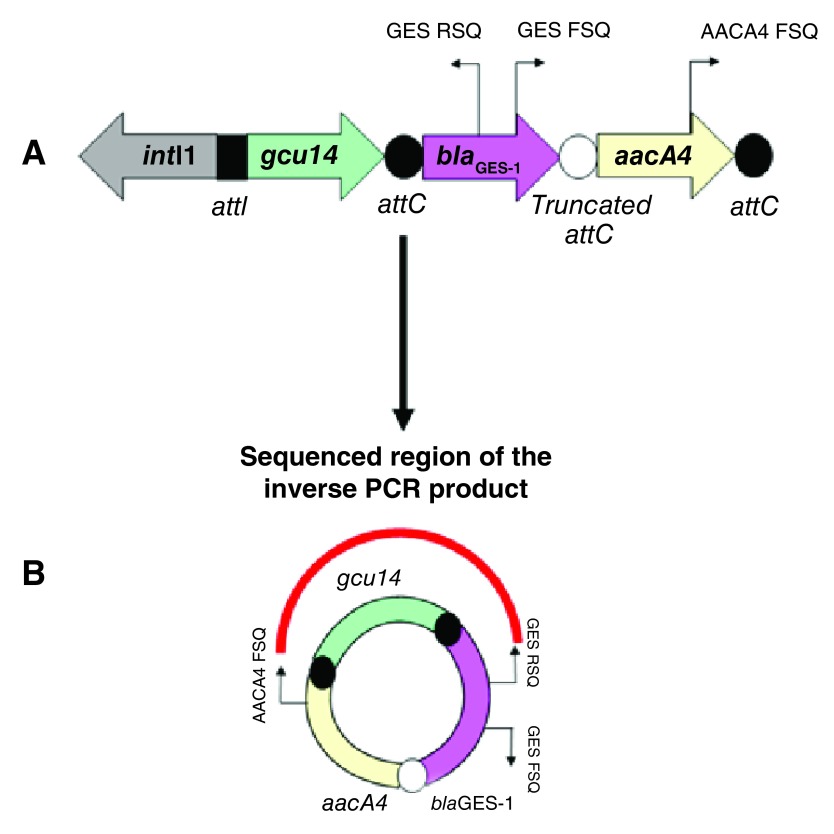
Schematic representation of the free circular form cassette array resulted from the inverse PCR assay. ( **A**) inserted/linearized form of integron harbouring the fused cassettes. Arrows show the gene transcription orientation. Thin arrows represent the annealing sites of the inverse PCR and sequencing primers whose generated product corresponded to the cassette circular form illustrated in ( **B**). ( **B**) Illustration of the free circular form of gene cassettes represented in ( **A**). Thin arrows show the primers used to obtain the inverse PCR product (GES FSQ and GES RSQ) and the primers used in sequencing (AACA4 FSQ and GES RSQ) that revealed the excision in block of the entire gene cassette arrangement ( *gcu14*- *bla*_GES-1_/ *aacA4*) from integron (red curved line).

Positive results were obtained for the *gcu14*, *gcu14*- *aacA4* and *gcu14*- *bla*_GES-1_/ *aacA4* circular forms, but not for *bla*_GES-1_ and *aacA4* alone. This finding indicates that the GES-1 *attC* is not functional and that the fused gene cassette is excised as a unique cassette. Moreover, the presence of *gcu14*- *aacA4* circular form suggests that this strain carries a second integron containing this gene cassette arrangement. Sequencing assessed the recombination point where excision occurred, confirming the occurrence of free circular forms ( Figure 3). This is in agreement with the presence of the PcW configuration, in which the corresponding *intI1* gene codes for a high efficient integrase ^11^. The lack of activity of a truncated *attC* had also been observed before when associated with *aadA10* ^13^. However, Ramirez and colleagues ^14^ showed that the integrase was able to recognize and mediate excision of a truncated site associated to *aadA1*, indicating that the genetic context of such truncated sites could influence their role in IntI1 recognition and mobilization.

**Figure 4.**
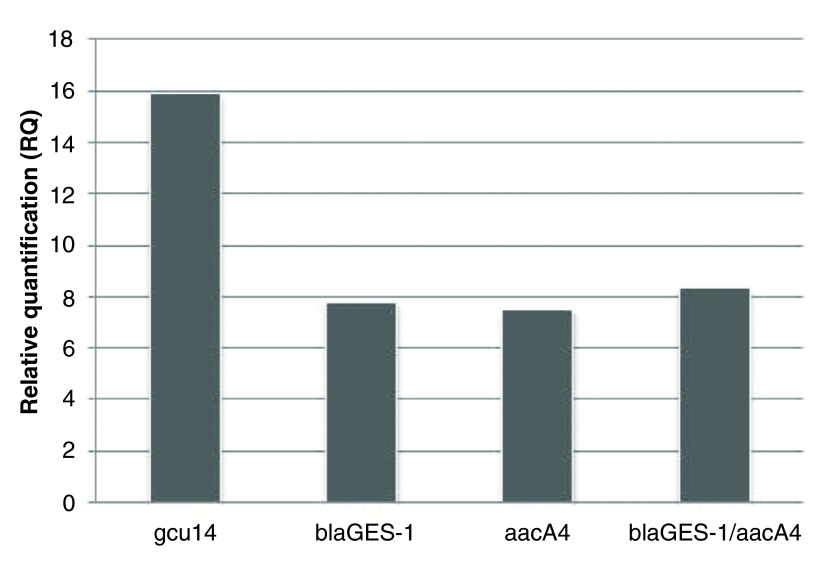
Transcription level of *gcu14*- *bla*_GES-1_/ *aacA4* gene cassette array. The relative quantification values obtained by real time RT-PCR are indicated for each gene cassette and for the fusion *bla*_GES-1_/ *aacA4* when considered as a unique gene.

The relative quantification performed by real time RT-PCR revealed that PS1 and PS26 presented very similar RQ values for *gcu14*- *bla*_GES-1_/ *aacA4* transcription ( Figure 4). This result was expected since integrons from these two strains have the same backbone, including the Pc promoter, and are at the same genetic environment ^10^.

*gcu14*, the first cassette in integrons with the weak PcW configuration, presented approximately two-fold higher transcription when compared to *bla*_GES-1_ and *aacA4* separately or when the fused cassette *bla*_GES-1_/ *aacA4* was considered ( Figure 4). The same RQ value obtained for *bla*_GES-1_, *aacA4* and the fusion reveals that these two ORFs are transcribed as a unique gene. The lower transcript amount of *bla*_GES-1_/ *aacA4* compared to *gcu14* lies on the distance between these gene cassettes and Pc, which is one of the determinants influencing cassette transcription ^1, 7^, and it shows the effect of cassette position on expression levels.

A putative promoter for *gcu14* (-35 TTGATG [17 bp] -10 TGTTAC) was found 45 bp upstream from its start codon, which has the potential to influence transcription. Moreover, the ORF-11, which enhances the translation efficiency of downstream TIR-deficient cassettes inserted in integrons ^5^, was found at the *attI1* region preceding the TIR-deficient *gcu14* gene cassette. This ORF contained its own Shine-Dalgarno (SD) sequence placed 8 bp upstream of the ATG codon. The ribosome at the ORF-11 stop codon could, therefore, be carried along the mRNA by lateral diffusion, reinitiating translation at the *gcu14* start codon. A potential SD sequence was identified 10 bp upstream of the fused cassette *bla*_GES-1_/ *aacA4*. In addition, the loss of the GES-1 *attC* region, which is involved in stem-loop formation, may enhance the chances of *aacA4* translation, since this *attC,* reduced to the 6bp of the 1L core site, no longer constitutes a physical barrier to ribosome progression ^4^. Together, these findings create a scenario for the occurrence of *gcu14*- *bla*_GES-1_/ *aacA4* expression in PS1 and PS26, which then provides a possible explanation for their resistance profile to β-lactams and aminoglycosides that has been observed elsewhere ^10^.

RQ fused cassette raw data for Pseudomonas aeruginosa isolates PS1 and PS26 The first column describes the P. aeruginosa isolates (PS1 and PS26) and the gene targeted in the quantitative PCR for measuring their transcription (gcu14; GES-1; aacA4; and the fusion GES-1-aacA4 and rpsL), which was performed in triplicate. Also in this column are the negative controls for each PCR reaction (NTC). The second column displays the cycle threshold (CT), i.e., the PCR cycle in which the fluorescence was detected by the machine. The third and the fourth columns refer to the standard deviation and the average of CT values, respectively, between triplicates. The fifth column refers to the to the normalization of the target gene transcript amount relative to that of endogenous gene (rpsL). The sixth column corresponds to the results of relative quantification. For example, the gcu14 gene and GES gene in PS1 had RQ values of 15,88 and 7,80. It means that gcu14 is 2-fold more transcribed than GES in PS1 isolate. Click here for additional data file. Copyright: © 2015 Fonseca ÉL and Vicente ACP 2015 Data associated with the article are available under the terms of the Creative Commons Zero "No rights reserved" data waiver (CC0 1.0 Public domain dedication).

## Conclusions

Fused cassettes have been found in class 1 integrons ^9, 13– 18^; however, the transcription of such structures has rarely been addressed. This work showed the transcription pattern of a fused cassette as a polycistronic mRNA and that these unusual structures are excised as a unique cassette. The mobilization in block of the entire *gcu14*- *bla*_GES-1_/ *aacA4* array together with its active transcription, and the presence of translational signatures demonstrate the potential for dissemination and expression of multidrug resistance, in a one-step fashion, to other bacteria. Therefore, such events could represent a threat to public health and to the establishment of efficient antibiotic regiments.

### Nucleotide sequence accession number

The sequence of the cassette array composed by the fusion has been deposited in the GenBank database under accession number DQ236170. The sequence obtained from the inverse PCR amplicon, showing the circular form gene organization, was submitted to GenBank under accession number KT336477.

## Data availability

The data referenced by this article are under copyright with the following copyright statement: Copyright: © 2015 Fonseca ÉL and Vicente ACP

Data associated with the article are available under the terms of the Creative Commons Zero "No rights reserved" data waiver (CC0 1.0 Public domain dedication). 

*Figshare:* Polycistronic transcription of fused cassettes and identification of translation initiation signals in an unusual gene cassette array from *Pseudomonas aeruginosa* doi: http://dx.doi.org/10.6084/m9.figshare.651717 ^19^
