# Supplementary material for: Polycistronic transcription of fused cassettes and identification of translation initiation signals in an unusual gene cassette array from Pseudomonas aeruginosa
Source: F1000Res. 2015 Nov 27;2:99. Originally published 2013 Mar 28. [Version 3] doi: 10.12688/f1000research.2-99.v3 (PMC4670013; doi:10.12688/f1000research.2-99.v3)
Supplement: Peer review history

# Peer review: Referee response for version 3

**DOI:** 10.5256/f1000research.8053.r11365

**Published:** 2015-11-30

**Authors:** Sally Partridge

**Affiliations:** 1 Sydney West Area Health Service, Sydney, NSW, Australia

The grey shading to indicate the attC site should not start until the start of 1L and the portion derived from attI1 is still not indicated.

I have read this submission. I believe that I have an appropriate level of expertise to confirm that it is of an acceptable scientific standard.

# Peer review: Referee response for version 2

**DOI:** 10.5256/f1000research.3720.r10226

**Published:** 2015-11-18

**Authors:** Sally Partridge

**Affiliations:** 1 Sydney West Area Health Service, Sydney, NSW, Australia

This manuscript has been improved and the points I raised have been addressed, but I have some comments about the new Fig. 2:

1. The underlined uppercase letters indicating the blaGES-1 coding region should extend until the stop codon at position 914.
2. ttagat of the IR site shown for the complete blaGES-1 attC is really part of the adjacent 3'-CS in AF355189 (most of the 1R core site belonging to the blaGES cassette (TTAGAC) is found at the start of the linear cassette). I would suggest ending this sequence after the first g of this site.
3. The partial 1L of the truncated blaGES cassette (924-9), the nucleotides derived from the attI1 site (930-6) and 1R of the aacA4 cassette (937-42) should be indicated. I would suggest ending the sequence of the truncated cassette after 1R of aacA4, as the alignment of the complete attC with the start of the aacA4 cassette beyond this point is not really useful.

I have read this submission. I believe that I have an appropriate level of expertise to confirm that it is of an acceptable scientific standard, however I have significant reservations, as outlined above.

# Response

**Published:** 2015-11-19

**Authors:** Erica Fonseca

**Affiliations:** Instituto Oswaldo Cruz, Brazil

Reviewer: The underlined uppercase letters indicating the blaGES-1 coding region should extend until the stop codon at position 914.

Response: Reviewer is right. This was corrected in the figure. Actually, the figure was modified in order to make clearer the differences between the canonical GES-1 attC and the truncated GES-1 attC found in this study.

Reviewer: ttagat of the IR site shown for the complete blaGES-1 attC is really part of the adjacent 3'-CS in AF355189 (most of the 1R core site belonging to the blaGES cassette (TTAGAC) is found at the start of the linear cassette). I would suggest ending this sequence after the first g of this site.

Response: We agree and this was corrected in the new figure.

Reviewer: The partial 1L of the truncated blaGES cassette (924-9), the nucleotides derived from the attI1 site (930-6) and 1R of the aacA4 cassette (937-42) should be indicated. I would suggest ending the sequence of the truncated cassette after 1R of aacA4, as the alignment of the complete attC with the start of the aacA4 cassette beyond this point is not really useful.

Response: We agree and this was modified in the new figure.

# Peer review: Referee response for version 2

**DOI:** 10.5256/f1000research.3720.r10563

**Published:** 2015-09-28

**Authors:** Béatrice Berçot

**Affiliations:** 1 Service de Bactériologie-Virologie, Hôpital de Bicêtre, Paris, France

I have read the updated paper which is clear and well written. Little is known in transcription of fused cassettes and these experimental data highlighted that the two genes are cotranscripted and mobilized together. The author answered all my request and I have  not supplementary remarks

I have read this submission. I believe that I have an appropriate level of expertise to confirm that it is of an acceptable scientific standard.

# Peer review: Referee response for version 1

**DOI:** 10.5256/f1000research.903.r961

**Published:** 2013-05-21

**Authors:** Béatrice Berçot

**Affiliations:** 1 Service de Bactériologie-Virologie, Hôpital de Bicêtre, Paris, France

This work is conducted to address the transcription of the fused gene cassette *bla*_GES-1_/ *aacA4.* This arrangement is rare but not exceptional in a class 1 integron. In this work, the experimentation confirmed that the three gene cassettes harbored in this class 1 integron were expressed together from a unique polycistronic transcript.

It seems that the authors have observed a circular form containing the *gcu14* gene cassette associated with the *aacA4* gene cassette. Could they explain this arrangement? Is it possible that the *Pseudomonas* isolates harbored another class 1 integron containing the *gcu14* gene followed by the *aacA4* gene?

The authors have to provide in the paper the classical length of the *bla*_GES-1_ *attC *site, which is 110bp and contained the 1L, 2L, 2R and 1R sequences-shown in bold. 

TTGACGCCC **GTCTAAC** ****AATTC **GTTCAAGC** ****CGACGTTGCTTCGTGGCGGCGCTTGCGTGCTACGCTAAGCTTCGCACGCCGCTTGCCACTGCGCACCGCG **GCTTAAC** ****TCAGGC **GTTAGNC**

In the *Pseudomonas *SP26 and PS1 isolates, the *bla*_GES- 1_ *attC* site is reduced to the 6 bp of 1 L box.

TTGACGCCC **GTCTAA** ****AACAAAGTTAGGCATCACAAAGTACAGCATC ***GTG***

So, the distance from of the stop of *bla*_GES-1_ to the start codon of the *aacA4 *gene is 46bp. The translational start codon of *aacA4* gene is erroneous in the Genbank data base ( DC236170). Indeed, it has been determined by N-terminal amino acid sequencing to be a GTG (in italic in the sequence above) and the beginning of the cassette was 24 bp before [ Hanau-Bercot B. et al., 2002].

-Could the Genbank annotation be modified as explained above?

-The Appendex is not necessary and should be deleted.

-In the sentence page 4, line 13, “The single copy of *rpsL* gene of *Pseudomonas*...”, should be replaced by “the *rpsL* gene of *Pseudomonas* (see Bodilis et al., 2012)

-In the conclusion, page 6, change “never” to “rarely”.

I have read this submission. I believe that I have an appropriate level of expertise to confirm that it is of an acceptable scientific standard, however I have significant reservations, as outlined above.

# Response

**Published:** 2015-08-05

**Authors:** Erica Fonseca

**Affiliations:** Instituto Oswaldo Cruz, Brazil

It seems that the authors have observed a circular form containing the gcu14 gene cassette associated with the aacA4 gene cassette. Could they explain this arrangement? Is it possible that the Pseudomonas isolates harbored another class 1 integron containing the gcu14 gene followed by the aacA4 gene?

**Response:** Yes, the presence of the gcu14-aacA4 circular form indicates that this strain harbours a second integron composed by the gcu14-aacA4 arrangement. However, this result does not invalidate our main conclusion, which is that the truncated GES-1 attC site is not functional and this gene is only mobilized when recombination occurs in aacA4 attC. A brief explanation was included in the text (Results section).

The authors have to provide in the paper the classical length of the *bla*_GES-1_ *attC *site, which is 110bp and contained the 1L, 2L, 2R and 1R sequences-shown in bold.

**Response:** A figure showing the truncated and the complete canonical *attC* from GES1 was included in the new version.

So, the distance from of the stop of blaGES-1 to the start codon of the aacA4 gene is 46bp. The translational start codon of aacA4 gene is erroneous in the Genbank data base (DC236170). Indeed, it has been determined by N-terminal amino acid sequencing to be a GTG (in italic in the sequence above) and the beginning of the cassette was 24 bp before [Hanau-Bercot B. et al., 2002].

-Could the Genbank annotation be modified as explained above?

**Response:** We performed this modification and the updated sequence regarding the beginning of aacA4 gene is already accessible on GenBank database under the same accession number (DQ236170).

-The Appendex is not necessary and should be deleted.

**Response:** The appendix is only for helping referees in their evaluation. It will not be published.

-In the sentence page 4, line 13, “The single copy of rpsL gene of Pseudomonas...”, should be replaced by “the rpsL gene of Pseudomonas (see Bodilis et al., 2012).

**Response:** this was modified in the new version of the manuscript.

In the conclusion, page 6, change “never” to “rarely”.

**Response:** this was modified in the new version of the manuscript.

# Peer review: Referee response for version 1

**DOI:** 10.5256/f1000research.903.r920

**Published:** 2013-05-02

**Authors:** Sally Partridge

**Affiliations:** 1 Sydney West Area Health Service, Sydney, NSW, Australia

This paper looks at expression of genes in a gene cassette array that includes a fused cassette and at the excision of cassette from this array. Polycistronic transcripts from cassette arrays and the excision of other fused cassettes have previously been noted by others, as cited in this paper. The sequences in Appendix 1 need to be annotated properly, rather than just the results of searches given, and the point illustrated by each sequence needs to be explained to make it possible to assess whether they support the conclusions drawn in the paper. 

Some reorganisation (moving some information in the Results and Discussion to the Introduction) would help to make the paper easier to follow and the Conclusions section is very short and could be expanded. The English could also be improved and the manuscript checked for typos etc. 

A few specific points also need correcting or clarifying:

- Abstract Line 5- fused cassettes do not always have fused orfs (e.g. *aacA1*/ *orfG*)
- Abstract 6th Line from end and Results and Discussion p. 5 1st Line of 2nd paragraph – what is in the *gcu14*- *aacA4* circular form? Should this be *bla*GES-1/ *aacA4*?
-  Materials and Methods Line 1 – this needs rewording to explain that gcu is a gene cassette of unknown function.
- Results and Discussion p. 4 – the fusion here is the type where part of the *bla*GES-14 attC site is replaced by part of the *attI1* site (see ref. 14). The LH and RH domains are part of the *attC* site, rather than flanking it. The 5′ UTR contains 6 bp of the *attC* site.
-  Results and Discussion p. 5 – most of the 1L core site and the 1R core site of the *bla*GES *attC* site are present. 
-  Results and Discussion p. 6 – the deletion of part of the *attC* site doesn’t bring the *aacA4* gene much closer to Pc and ref. 14 makes slightly different point (that expression of the downstream cassette may be enhanced).

Formatting “14” of *gcu14* and “A4” of *aacA4* should be in italics.Transposon, gene and species names etc are not correctly formatted in the references.

I have read this submission. I believe that I have an appropriate level of expertise to confirm that it is of an acceptable scientific standard, however I have significant reservations, as outlined above.

# Response

**Published:** 2015-08-05

**Authors:** Erica Fonseca

**Affiliations:** Instituto Oswaldo Cruz, Brazil

The sequences in Appendix 1 need to be annotated properly, rather than just the results of searches given, and the point illustrated by each sequence needs to be explained to make it possible to assess whether they support the conclusions drawn in the paper.

**Response:** The sequence resulted from inverse PCR product, showing the circular form of the gcu14-blaGES-1-aacA4 array, was submitted to GenBank under accession number KT336477. This information was included in the text. Moreover, we provided a new figure showing a schematic representation of primer targeting sites used in inverse PCR in order to make easier for the reviewers comprehend our strategy and results.

Some reorganisation (moving some information in the Results and Discussion to the Introduction) would help to make the paper easier to follow and the Conclusions section is very short and could be expanded. The English could also be improved and the manuscript checked for typos etc.

**Response:** We agree that some points from results and discussion section would be more adequate in the introduction section, and they were moved in the new version of the manuscript (Page 3, lines 59-60 and 65-70; page 4, lines 71-76 in the new version).

The English was reviewed by a native spoken-English, and the conclusion was expanded.

A few specific points also need correcting or clarifying:

Abstract Line 5- fused cassettes do not always have fused orfs (e.g. aacA1/orfG)

**Response:** We agree and it was modified: We affirmed that only in some cases fused orfs can be created.

Abstract 6th Line from end and Results and Discussion p. 5 1st Line of 2nd paragraph – what is in the gcu14-aacA4 circular form? Should this be blaGES-1/aacA4?

**Response:** The presence of the gcu14-aacA4 circular form indicates that this strain harbours a second integron composed by the gcu14-aacA4 arrangement. However, this result does not invalidate our main conclusion, which is that the truncated GES-1 attC site is not functional and this gene is only mobilized when recombination occurs in aacA4 attC. A brief explanation was included in the text (Results section).

     Materials and Methods Line 1 – this needs rewording to explain that gcu is a gene cassette of unknown function.

**Response:** It was included in the text.

    Results and Discussion p. 4 – the fusion here is the type where part of the blaGES-1 attC site is replaced by part of the attI1 site (see ref. 14). The LH and RH domains are part of the attC site, rather than flanking it. The 5′ UTR contains 6 bp of the attC site.

**Response:** We change the text emphasizing that the attC was replaced by part of attI1.

 The referee is right; LH and RH domains are part of the attC site, rather than flanking it. This general idea concerning attC site and its domains was modified throughout the text.

     Results and Discussion p. 5 – most of the 1L core site and the 1R core site of the blaGES attC site are present.

**Response:** In fact, only the 1L core site is present in the truncated attC. We included a figure (figure 2) in this new version showing this.

     Results and Discussion p. 6 – the deletion of part of the attC site doesn’t bring the aacA4 gene much closer to Pc and ref. 14 makes slightly different point (that expression of the downstream cassette may be enhanced).

**Response:** The referee is right. This statement was removed.

Formatting “14” of gcu14 and “A4” of aacA4 should be in italics. Transposon, gene and species names etc are not correctly formatted in the references.

**Response:** All formatting errors were properly corrected.
